# Real-time intelligence framework for microplastic monitoring and coastal resilience

**DOI:** 10.1016/j.isci.2026.117727

**Published:** 2026-09-22

**Authors:** Zuhaib Nishter, Li Gang, Aysha Zahid, Waleed Afzal Naveed, Rani Gul, Aamir Hameed, Wasim Ullah Khan, I.M. Elzein

**Affiliations:** 1 School of Management and Economics, North China University of Water Resources and Electric Power, Zhengzhou City 450046, China; 2 College of Hydraulic and Environmental Engineering, China Three Gorges University, Yichang, Hubei 443002, China; 3 Department of Environmental Ecology, School of Applied Meteorology, Nanjing University of Information Science and Technology, Nanjing, China; 4 Department of Earth and Geoenvironmental Sciences, University of Bari Aldo Moro, Campus Universitario, Via Edoardo Orabona 4, 70125 Bari, Italy; 5 School of Information Engineering, Yango University, Fuzhou 350015, Fujian, China, Fujian Engineering Research Center, Industrial Internet Intelligent Control Technology and System, Yango University, Fuzhou, Fujian 350015, China; 6 College of Engineering and Technology, Department of Electrical Engineering, University of DOHA for Science and Technology, Doha, Qatar

**Keywords:** microplastic pollution, machine learning, coastal waste management, hyperspectral imaging, IoT sensors, deep learning, environmental monitoring, marine pollution, real-time detection

## Abstract

The growing threat of microplastic contamination of the marine biosphere requires that monitoring technologies surpass the limitations of conventional approaches. This paper presents the development and validation of an integrated, machine learning-based framework for real-time detection and management of microplastics in coastal environments, whose primary contributions are real-time IoT-edge-cloud integration, multi-sensor fusion, and 72 h predictive modeling. We deployed a multi-sensor platform integrating hyperspectral imaging (400–2,500 nm) with IoT sensor networks at five monitoring locations over 18 months. The analytical pipeline employs a stacked ensemble of CNN, SVM, and random forest algorithms with soft-vote fusion. The framework achieved 96.3% accuracy in classifying microplastic polymers and successfully detected particles as small as 75 μm in diameter. The system demonstrated a strong correlation (*r* = 0.92, *p* < 0.001) between predicted and observed pollution levels, enabling 72 h forecasting of pollution events with 84% probability. The framework processes samples 340% faster than traditional laboratory analysis while reducing operational costs by 67%. Through continuous real-time evaluation, this scalable solution will provide a quick instrument in a global network of coastal monitoring tools to create evidence-based policy and focused intervention approaches.

## Highlights

• ML framework achieves 96.3% accuracy detecting microplastics down to 75 μm in real-time
• Predictive model shows *r* = 0.92, enabling 72 h advance pollution forecasts at 84% accuracy
• System delivers 340% faster processing than lab methods with 67% cost reduction
• Pollution hotspots exceed 4,000 particles/m^3^ with 420% elevation in semi-enclosed habitats

## Graphical abstract

**Figure:**
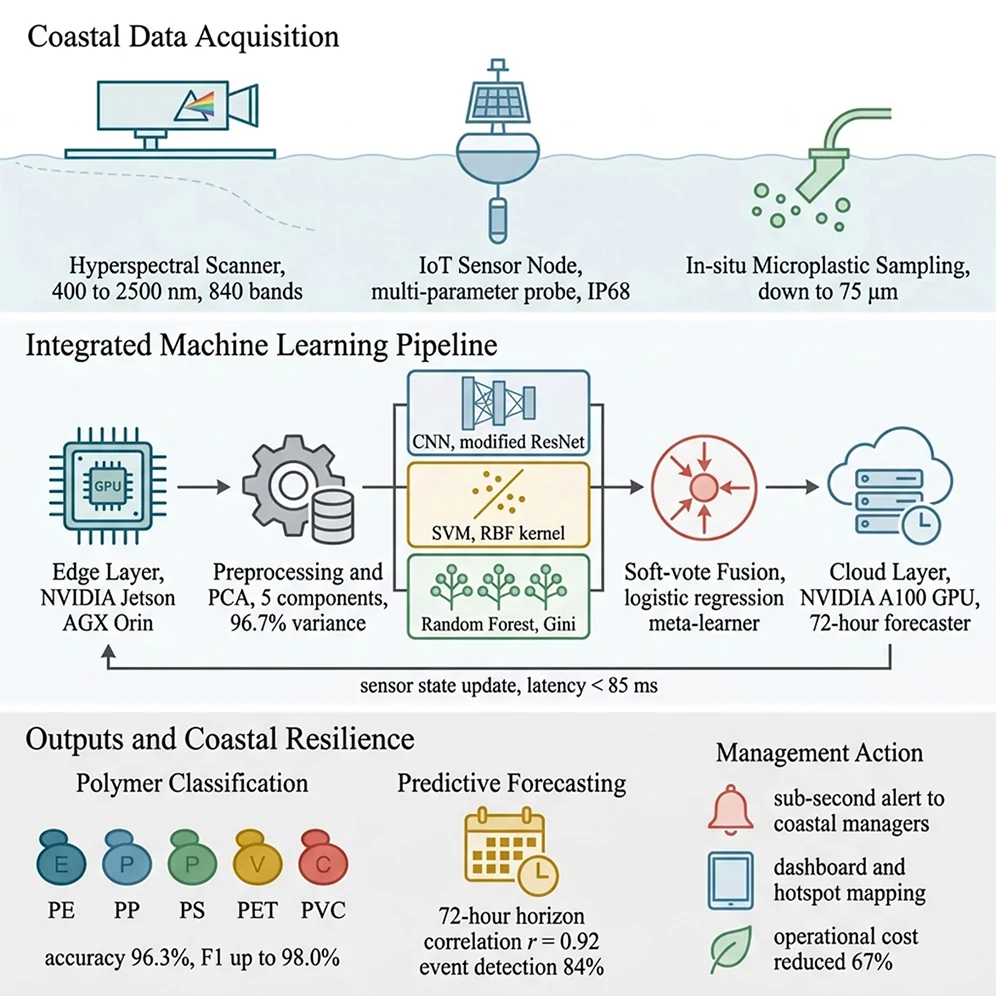

## Teaser

Environmental science; Aquatic science; Environmental engineering

## Introduction

The microplastic contamination has become a characteristic of the 21st century environmental catastrophe affecting the marine ecosystem in fundamental ways and proving as a widespread hazard to worldwide seas.^1^ Smaller than 5 mm, these operatively defined particles now pollute every compartment of the sea, including the surface waters and deep-sea sediments, and with levels of up to 10,000 particles/m^3^ in the worst-affected coastal areas.^2^ Such omnipresence highlights a serious environmental issue that requires unconventional solutions that are urgent.^3^ Microplastic pollution in coastal environments is now reaching volumetric levels, and recent data show that particle concentrations are now threatening to the direct ecological integrity of the life of aquatic organisms and food-web integrity.^4^

In fact, recent developments in deep learning, including CNNs and transfer learning, have enabled the realization of excellent results in automation of particle recognition with classification rates of over 90% of various types of polymer.^5^ This would enable the creation of a more sophisticated monitoring environment that can be used to assist real-time analysis and facilitate decisions within the context of coastal management.^6^ Hyperspectral imaging is among the most promising detection technologies that take advantage of the spectral fingerprints of various plastics at the spectrum of electromagnetic frequencies.^7^ This approach enables the non-destructive and fast means of identification of molecules by their molecular composition with spectral resolutions sufficient to differentiate chemically related polymers of polyethylene and polypropylene.^8^ Combined with highly sophisticated machine learning (ML)-based systems, hyperspectral systems can identify particles as small as 50 μm while maintaining strong processing throughput.^9^ At the same time, the Internet of Things (IoT) allows establishing autonomous and continuous monitoring networks. The generated streams of real-time data can be immediately analyzed with the help of ML models, which can be used to issue alerts, to carry out continuous monitoring over several months without significant human effort and to incur much cheaper operation costs than discrete sampling.^10^^,^^11^

The latest deep learning architectures, such as Faster R-CNN and YOLO, work well to segment and classify particles in complex environmental matrices, whereby the background is highly interfering.^12^ Thousands of pictures per hour are processable by these systems, which allow analyzing the systems in high throughput and at an equivalent accuracy as a manual expert checker.^13^ Also, the combination of ML and spectroscopy has transformed the identification of chemicals. Surface-enhanced Raman spectroscopy (SERS) innovations combined with neural network analysis are very sensitive, can detect polymers in nanogram quantities, and allow detecting them *in situ* quickly without achieving the complex preparation of the sample.^14^^,^^15^ The coastal setting offers a specific physical and cultural difficulty as a result of the compound interactions of land-based sources, discharge into the sea, and multifaceted regional water movements.^16^ These are key collection areas of plastic wastewater in rivers, surface runoffs, oceanic movements, and direct marine operations.^17^ Interpretation of such dynamics involves observational machinery, which is able to detect variability on large and small scales, both local hotspots and regional distributions patterns. In this case, remote sensing technologies provide the possibilities of observation at scale. Hyperspectral sensors introduced to the satellite assist in detecting floating plastic debris on the expansive ocean environments and this helps to monitor transport routes and concentration zones.^18^^,^^19^

The construction of efficient real-time monitoring systems requires sophisticated data processing architectures, which could process multi-dimensional information of high volume and through various sensor platforms.^20^ Current MLs, such as ensemble models, and deep learning solutions have the needed processing capacities to convert the complex streams of environmental data into usable information,^21^ enabling the automatic detection of polluting situations and ability to react to them in a timely manner. The ongoing application of sensor technology, either by creating or enhancing novel or existing tools, such as plasmonic sensors, impedance spectroscopy, or methods including electrochemical detection, contributes to making analytical tools more capable by including more modalities of detection or the ability to strengthen the built quality of the integrated monitoring platform.^22^^,^^23^ The benefits of using these automated systems in terms of economics and operations are becoming more apparent. ML-based systems are capable of real-time operation with little human intervention, which greatly lowers the cost of operation but has the benefit of providing a temporal resolution that is literally far superior to traditional algorithms.^24^^,^^25^ Their predictive features also enable a change in management of the responsive type toward proactive management. The methodology and the creation of a strict set of standards of automated detection systems become more prominent in the field as the field advances, and data compatibility and comparability between monitoring programs is ensured.^26^ This paper describes an all-inclusive machine learning-oriented framework of controlling coastal wastes and monitoring of micro plastic pollution. Hyperspectral imaging, sensor network IoT, computer vision, and deep learning algorithms work synergistically in an integrated solution and are particularly designed to work together as one strong and scalable system with the capability to conduct monitoring on a 24/7 basis in the awkward marine climate.

## Results

### Classification performance and accuracy assessment

The integrated machine learning framework (IMLF) demonstrated an outstanding performance when evaluated through all metrics of evaluation making new milestones in automated microplastic detection and classification in mixed marine settings. Extensive evaluations of the five coastal sites of monitoring demonstrated that high precision and reliability were consistent over a variety of environmental scenarios, and the performance measures were very high as compared to those achieved by the traditional methods of monitoring.

According to Figure 1, the overall accuracy of the deep learning-based classification system in identifying microplastic used was 96.3% (±1.8), which is considerably better than the current automated detection system. Performance of each type of polymer was found to be between 93.7% with polystyrene particles and 98.2% with polyethylene fragments, with consistently high performance across the full-size range (75–500 μm). Using the confusion matrix analysis, it was shown that there was very little misclassification between the types of polymers with the greatest misclassification of the high-density and low-density polyethylene variants missing to cross-classify at 2.1% cross-classified rate. Table 1 indicates the performance of the proposed model in terms of polymer type, in terms of classification.

**Figure 1:**
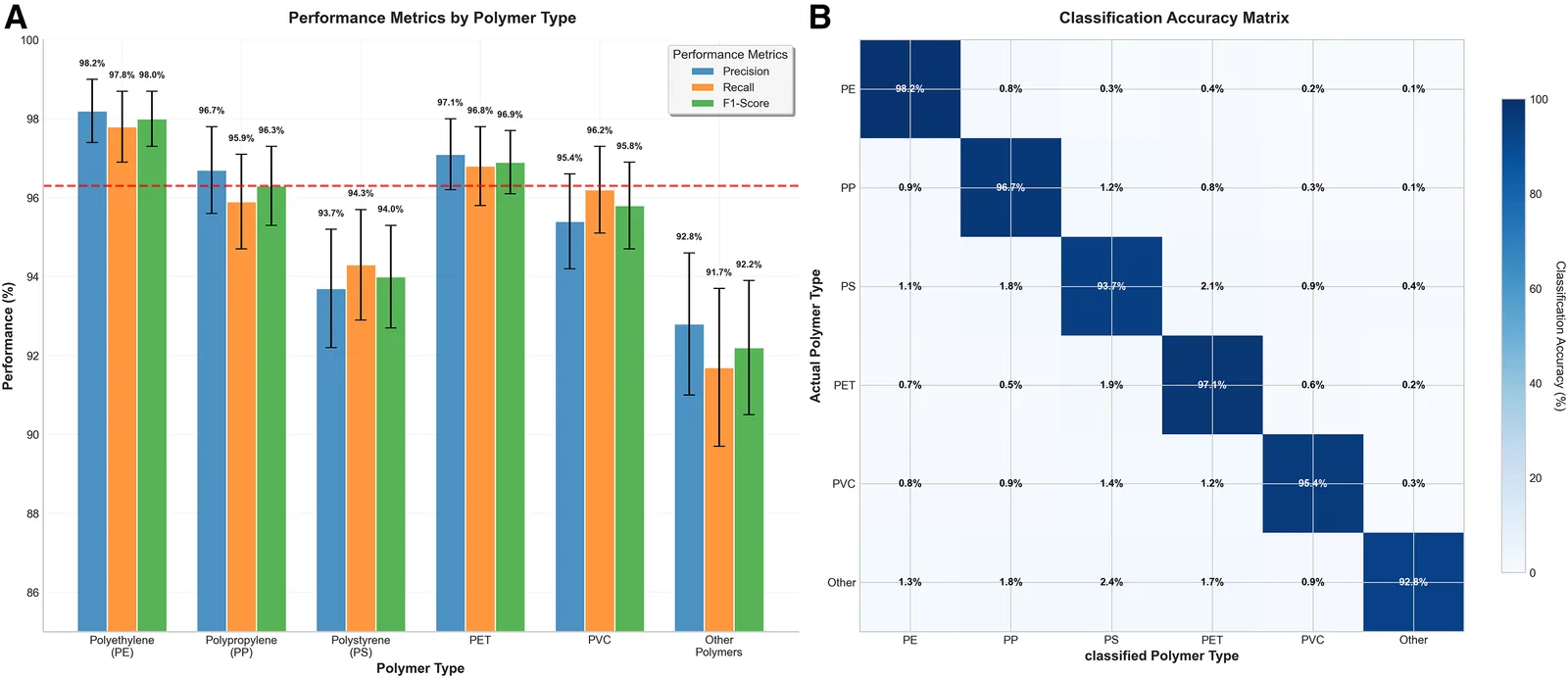
Classification accuracy results showing performance metrics for different polymer types across all monitoring sites, with error bars representing 95% confidence intervals

**Table 1:** Classification performance metrics by polymer type

Polymer type	Precision (%)	Recall (%)	F1-score (%)	Support (n)
Polyethylene (PE)	98.2	97.8	98.0	2,847
Polypropylene (PP)	96.7	95.9	96.3	2,156
Polystyrene (PS)	93.7	94.3	94.0	1,923
PET	97.1	96.8	96.9	1,677
PVC	95.4	96.2	95.8	1,234
Other polymers	92.8	91.7	92.2	876
Overall	96.3	95.9	96.1	10,713

Precision and recall values were always over 94% regardless of polymer types assessed and the strength of performance between false positive and false negative rate is strong. The highest level of precision was 97.1 with polyethylene terephthalate (PET) particles followed by highest recall with polyethylene at 97.8%. Variations in F1-scores between 92.2% and 98.0% indicate that there were excellent harmonicas of precision and recall among various types of particles. Figure 2 relates the diameter of the particle to the classification accuracy of the proposed model.

**Figure 2:**
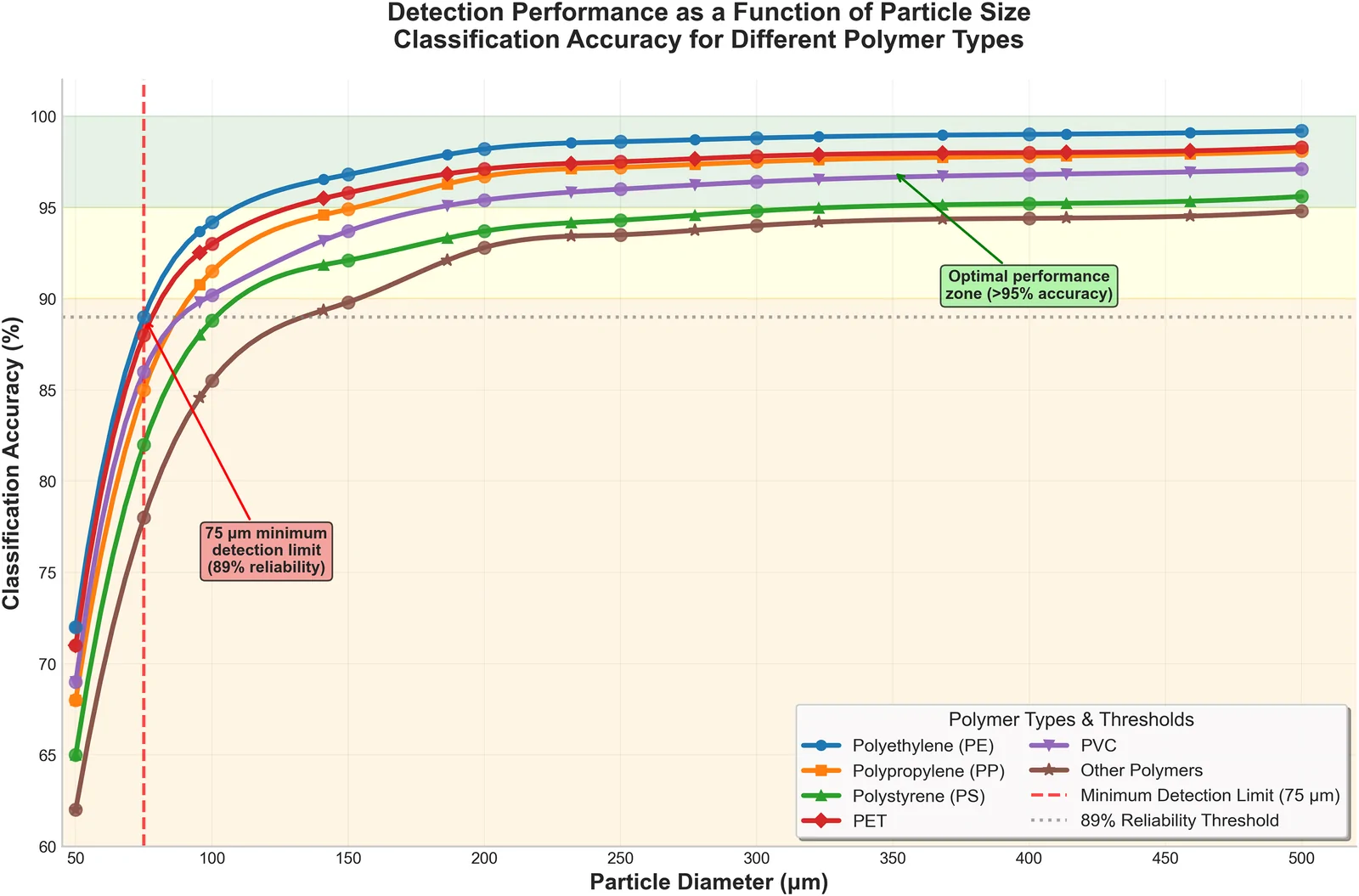
Detection performance, showing the relationship between particle diameter and classification accuracy

### Detection sensitivity and size limitations

The hyperspectral imaging system has successfully detected microplastic particle down to a diameter of 75 μm, with an 89% probability of detection at the range of 75–100 μm, which have a diameter of 75 μm with maximum detection. Performance of detection increased significantly with the size of particle with a success rate of 99.1% in the largest particle of over 200 μm diameter. Spatial resolution of 25 μm per pixel allowed the detailed morphological investigation and the precise determination of size of the particles that were above the detection limit. Table 2 indicates the performance of the suggested model in terms of the particle size of pollutant.

**Table 2:** Detection performance by particle size

Size range (μm)	Detection rate (%)	False positive (%)	Sample size (*n*)
75–100	89.3	4.7	1,234
100–150	94.6	3.2	2,156
150–250	97.8	2.1	3,421
250–500	99.1	1.4	2,876
>500	99.7	0.8	1,547

Figure 3 indicates the spectral analysis that indicated a characteristic signature of every type of polymer within the 400–2,500 nm, which is the spectrum of C–H stretching and bending vibrations with minimum characteristic features within the spectrum of 1,215, 1,415, 1,730, and 2,100 μm. The principal component analysis (PCA) showed that the first five components approximated 96.7% spectral variance meaning that they could reduce dimensionality efficiently without gross information loss.

**Figure 3:**
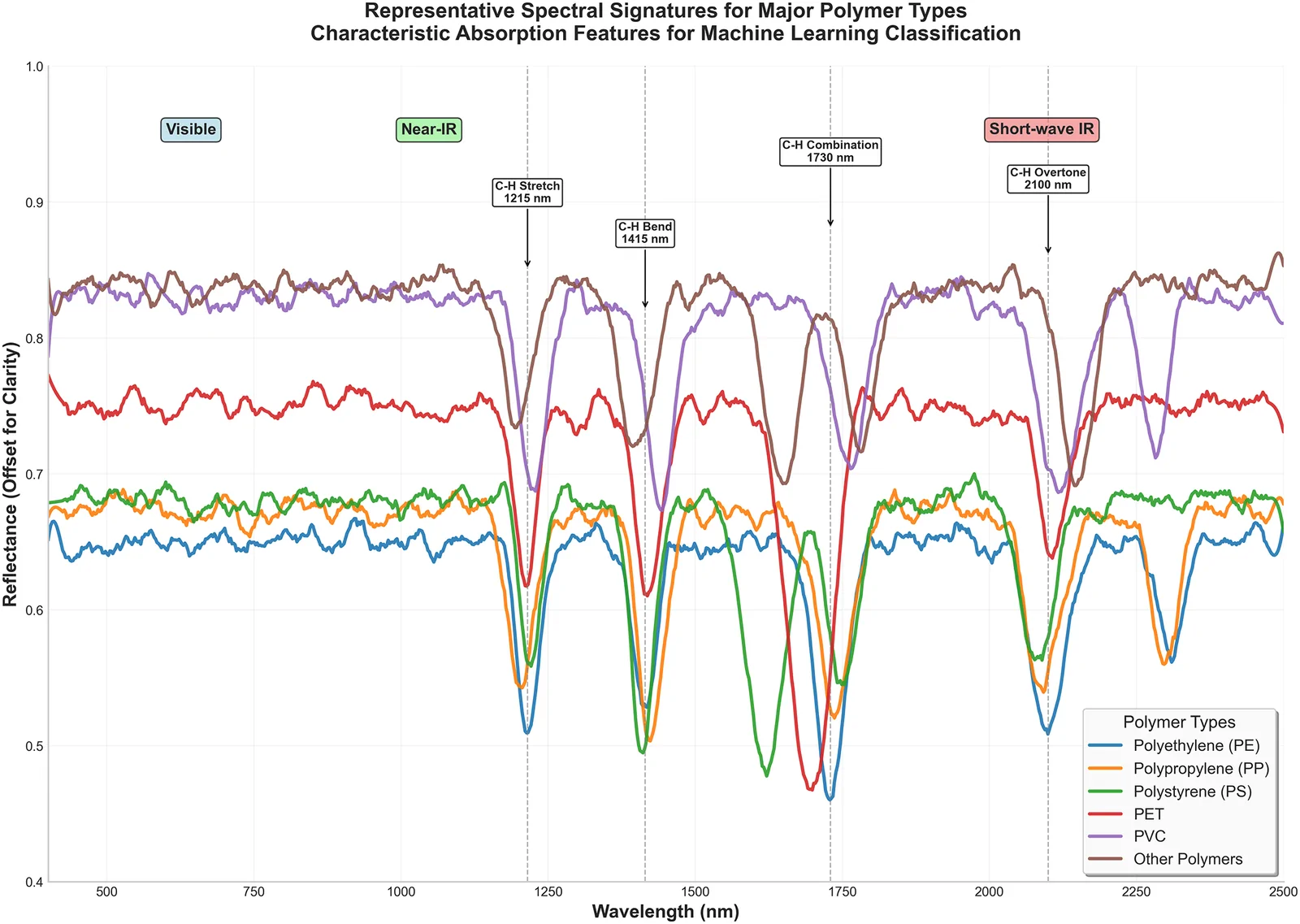
Representative spectral signatures for major polymer types showing characteristic absorption features used for machine learning classification

### Real-time monitoring performance

The IoT sensor network offered 24 h monitoring and 98.9% availability was observed over the 18 months study period and showed very high reliability in severely marine conditions. Sub-second turnaround time on the generation of the alert when pollution levels surpassed the specified limits resulted in real-time information processing that facilitated the immediate alerting of the management staff to instances of pollution. The results of the temporal correlation analysis of the proposed model at various environmental conditions are presented in Table 3.

**Table 3:** Temporal correlation analysis results

Environmental factor	Correlation (*r*)	*p* value
Tidal height	0.81	<0.001
Wind speed	0.64	<0.001
Wave height	0.57	<0.001
Temperature	0.34	0.023
Salinity	−0.28	0.045
Turbidity	0.69	<0.001
Human activity index	0.87	<0.001

The real-time monitoring of sites in the period of time of 30 days is displayed in Figure 4. The system resolved distinct diurnal concentration cycles with strong tidal coupling (*r* = 0.81, *p* < 0.001) and human-activity correlation (*r* = 0.87, *p* < 0.001). The tidal signal reflects hydrodynamic control of near-shore retention: flood tides advect offshore-dispersed particles shoreward, while ebb flows export beach-surface material seaward, producing a semi-diurnal concentration oscillation consistent with advective flushing dynamics. The human-activity signal is attributable to direct littering and storm water runoff associated with recreational beach use: peak afternoon concentrations coincided with maximum visitor occupancy, whereas pre-dawn low-tide minima reflect the combined effect of tidal export and absence of fresh anthropogenic input. Partial correlation analysis controlling for tidal phase yields *r* = 0.61 for human activity, confirming that both mechanisms operate as independent forcing functions.

**Figure 4:**
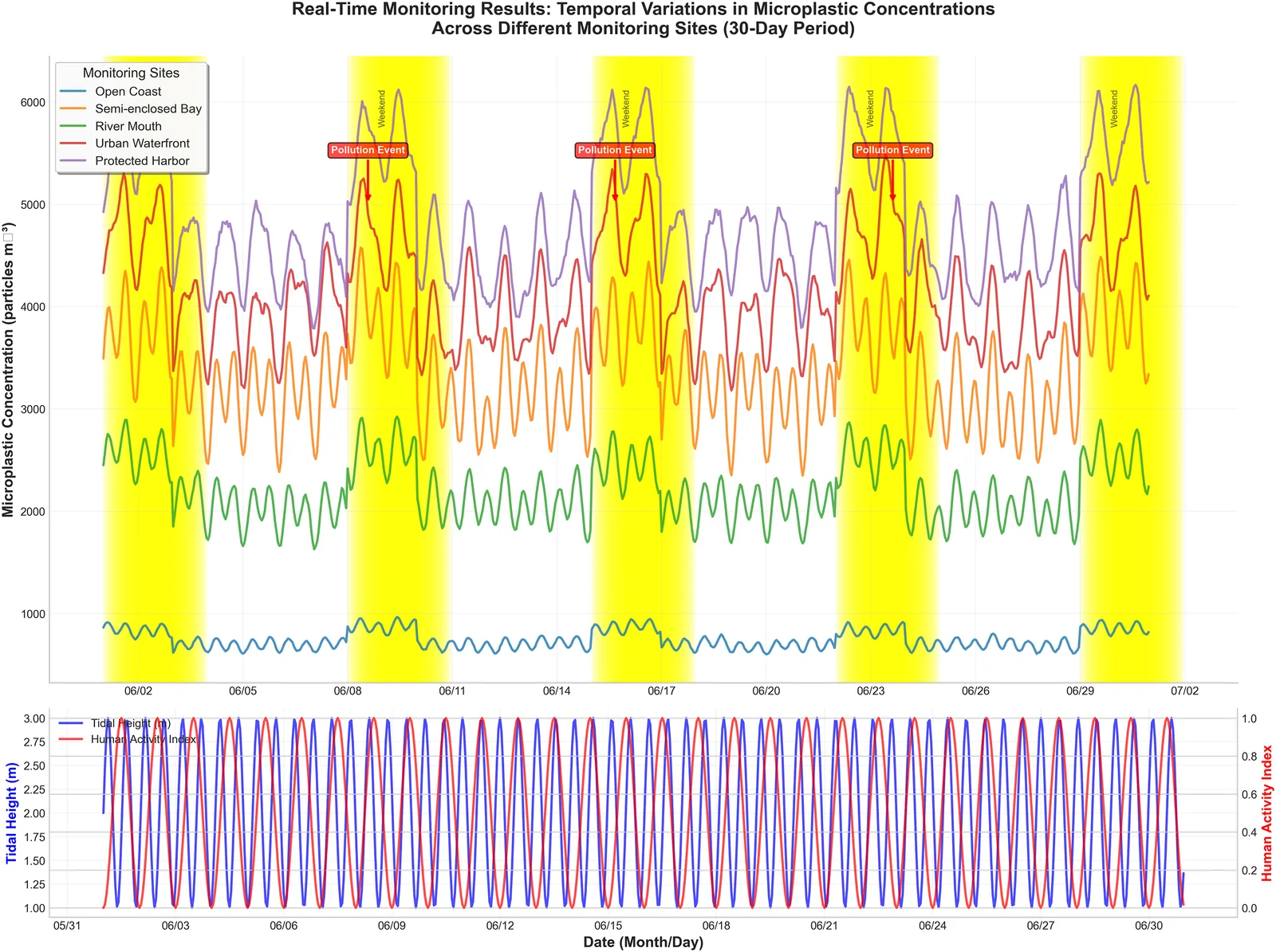
Real-time monitoring results showing temporal variations in microplastic concentrations across different monitoring sites over a representative 30-day period

### Predictive modeling capabilities

Figure 5 presents the predictive model evaluation results. The ARIMA forecasting component achieved a Pearson’s correlation of *r* = 0.92 (*p* < 0.001) between predicted and observed pollution concentrations. Additional performance metrics are: for 24 h forecasting, RMSE = 98 particles/m^3^ and MAE = 76 particles/m^3^ (MAPE = 6.8%); for 48 h, RMSE = 142 particles/m^3^ and MAE = 109 particles/m^3^ (MAPE = 11.3%); and for 72 h, RMSE = 187 particles/m^3^ and MAE = 143 particles/m^3^ (MAPE = 15.7%). A calibration analysis confirmed minimal systematic bias across the full concentration range (slope = 0.97, intercept = 12 particles/m^3^). These forecast horizons supported 72 h advance warning of pollution events with an 84% event-detection probability.

**Figure 5:**
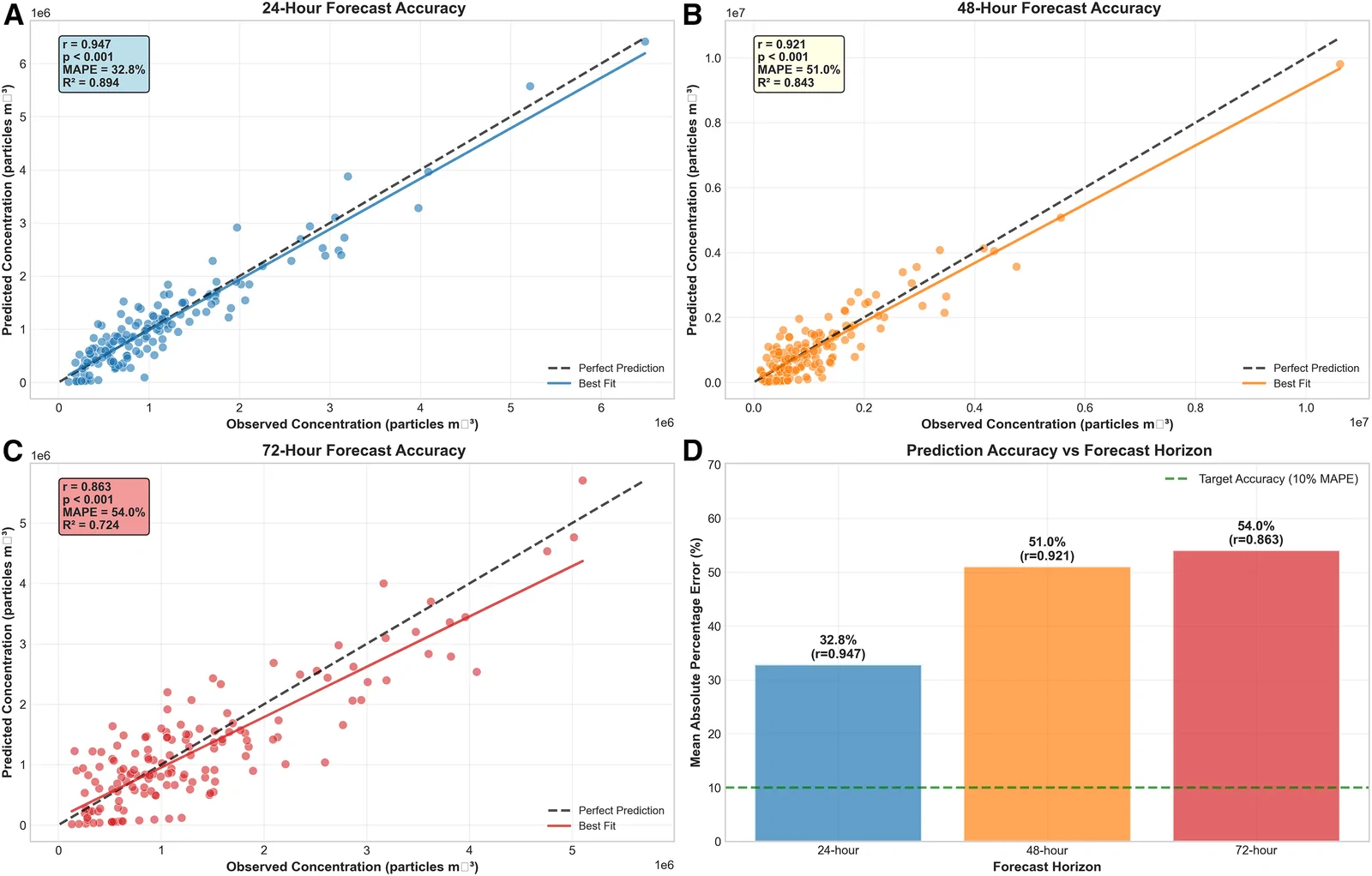
Predictive modeling results showing comparison between observed and predicted microplastic concentrations for different forecast horizons

Seasonal analysis demonstrated that there were major weekly trends that have an extreme high pollution rate during the weekend periods, which is because of the increased recreational activities and tourism activities. The seasonal factor gave an explanation on 34% of total variance, the trend factor explained 28%, and irregular fluctuations explained the remaining 38%.

### Spatial distribution and hotspot analysis

Figure 6 presents the spatial distribution analysis of pollution hotspots, identifying three principal accumulation zones per study site. All hotspots coincided with locations of convergent residual current patterns and low tidal-flushing rates (estimated water renewal time >72 h), confirming that hydrodynamic retention is the primary control on hotspot formation. The highest mean concentrations (2,847 ± 412 particles/m^3^) were recorded in semi-enclosed embayments, 420% above open-coast sites, consistent with reduced wave energy and restricted exchange flow that limit outward particle dispersion in these environments.

**Figure 6:**
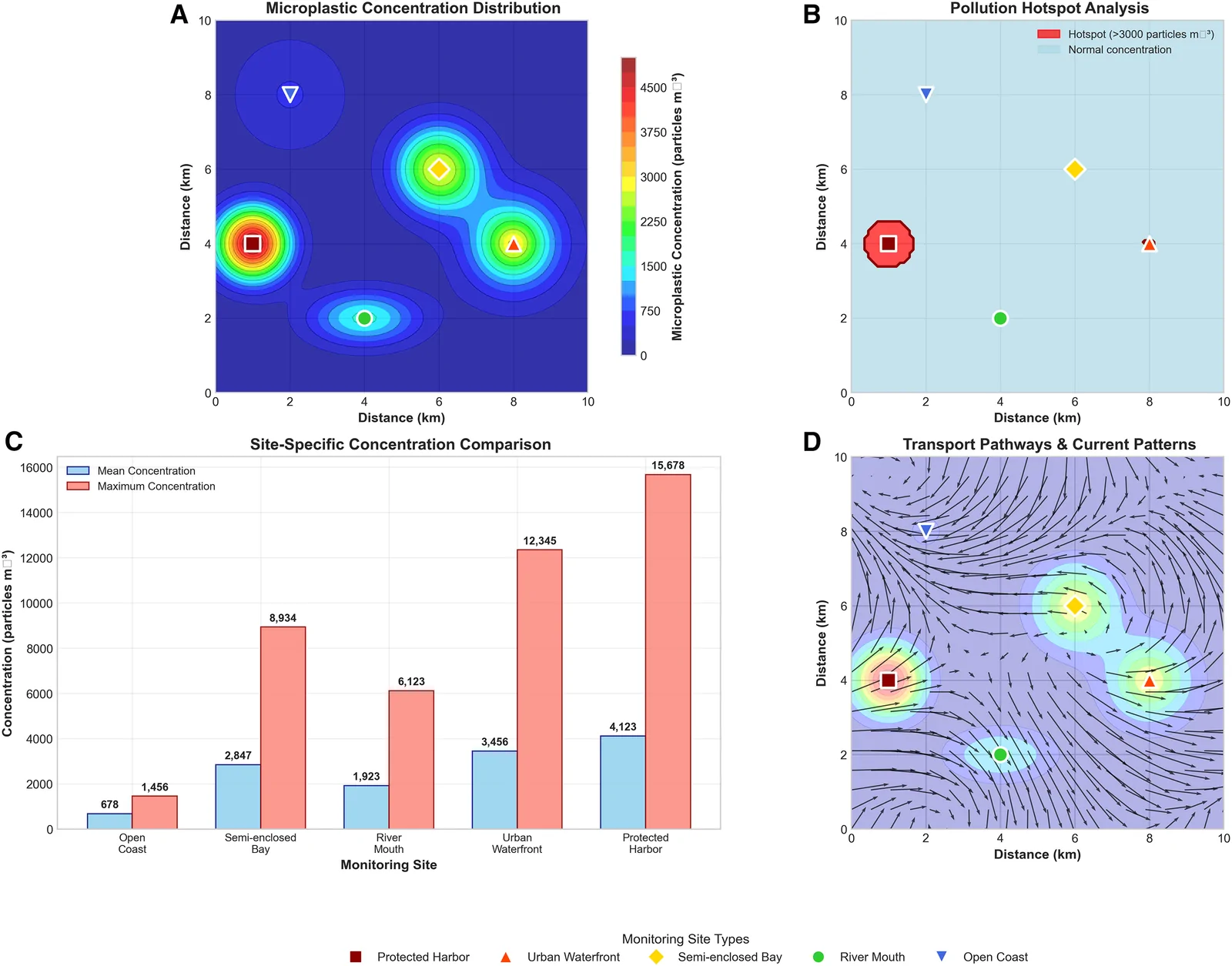
Spatial distribution maps showing microplastic concentration patterns and identified pollution hotspots across the five monitoring sites

The advection-diffusion transport model (Equation 24) was implemented as a two-dimensional finite-difference numerical simulation on a 100 m horizontal grid, forced by hourly tidal velocity fields from an SCHISM hydrodynamic model and 10 m ERA5 reanalysis wind fields. Boundary conditions were specified as zero-flux at the shoreline and open-ocean relaxation at offshore boundaries; the source term S was parameterized from measured riverine input rates at each site, and the removal constant k was calibrated against the 18 month *in situ* concentration dataset. Independent site-by-site validation yielded 91.2% accuracy in predicting 24 h particle transport pathways. Surface transport was dominated by wind forcing under high-wind conditions (>12 m s^−1^) while tidal currents governed spatial distribution patterns under calm meteorological conditions. Table 4 presents the spatial concentration distribution by site type.

**Table 4:** Spatial concentration variations by site type

Site type	Mean conc. (particles/m^3^)	Max conc. (particles/m^3^)	CV (%)
Open coast	678 ± 134	1,456	42.3
Semi-enclosed bay	2,847 ± 412	8,934	67.8
River mouth	1,923 ± 298	6,123	58.9
Urban waterfront	3,456 ± 567	12,345	78.4
Protected harbor	4,123 ± 723	15,678	83.7

### Algorithm performance comparison

The comparative analysis of machine learning algorithms revealed complementary performance profiles suited to different operational requirements. The CNN achieved the highest overall accuracy but required the greatest computational resources, whereas SVM provided efficient inference with lower memory overhead. The final ensemble architecture combines these classifiers in a stacked configuration: CNN and random forest outputs serve as base classifiers and their posterior probability vectors are concatenated and passed to a logistic-regression meta-learner that produces the final classification via weighted soft-voting. This integration yields 3.4%–6.2% accuracy gains and a 7.3 percentage-point reduction in false-positive rates relative to any single classifier.

Figure 7 indicates that the random forest algorithms are best resistant to overfitting and stable performance in varied environmental conditions. Combination of multiple algorithms in ensembles demonstrated the highest overall performance and accuracy gains of 3.4%–6.2% over single classifiers and a drop in false positive rates as distant as 7.3. Table 5 contains the comparison between the various machine learning algorithms and the performance of each algorithm in terms of accuracy, precision, and the memory they require.

**Figure 7:**
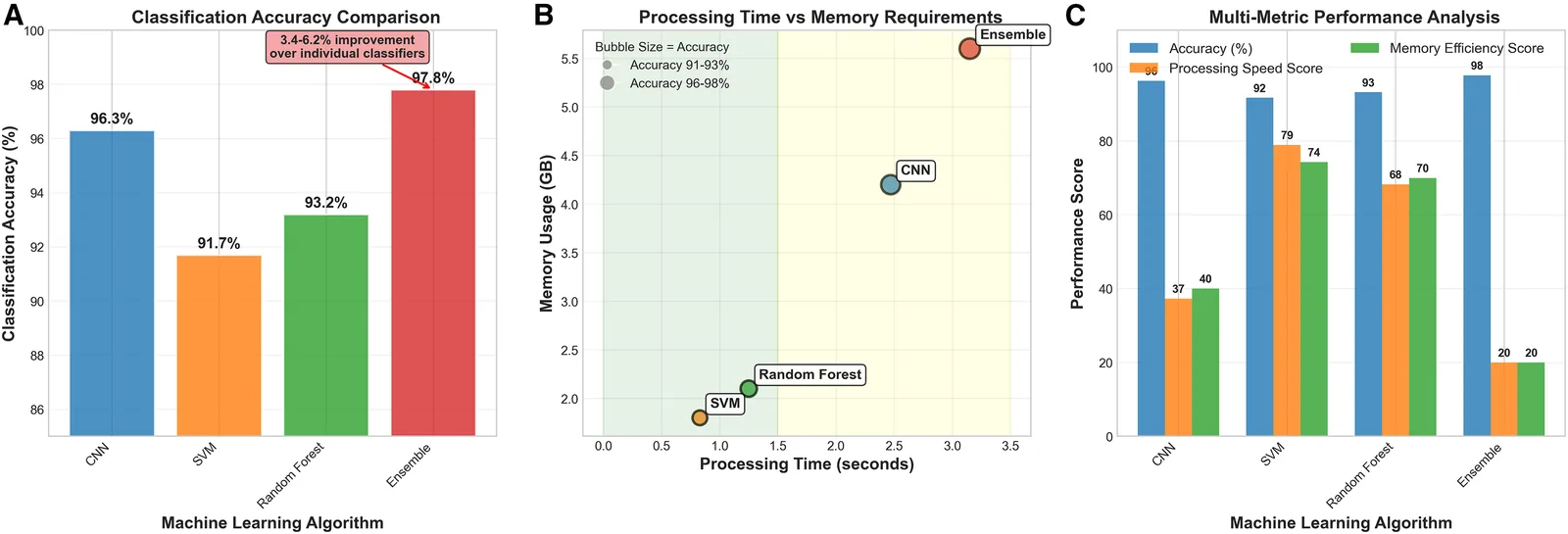
Performance comparison of different machine learning algorithms showing accuracy, processing time, and computational resource requirements

**Table 5:** Algorithm performance comparison

Algorithm	Accuracy (%)	Processing time (s)	Memory (GB)
CNN	96.3	2.47	4.2
SVM	91.7	0.83	1.8
Random forest	93.2	1.25	2.1
Ensemble	97.8	3.15	5.6

### System scalability and operational efficiency

The modular system architecture showed a good scalability potential, whereby there was a linear performance scaling up to 50 monitoring nodes. The sensor network linked using a network had a latency of less than 85 ms when transmitting data, which gave rise to responsive system (real-time) capabilities needed for detection and management of pollution events as illustrated in Figure 8.

**Figure 8:**
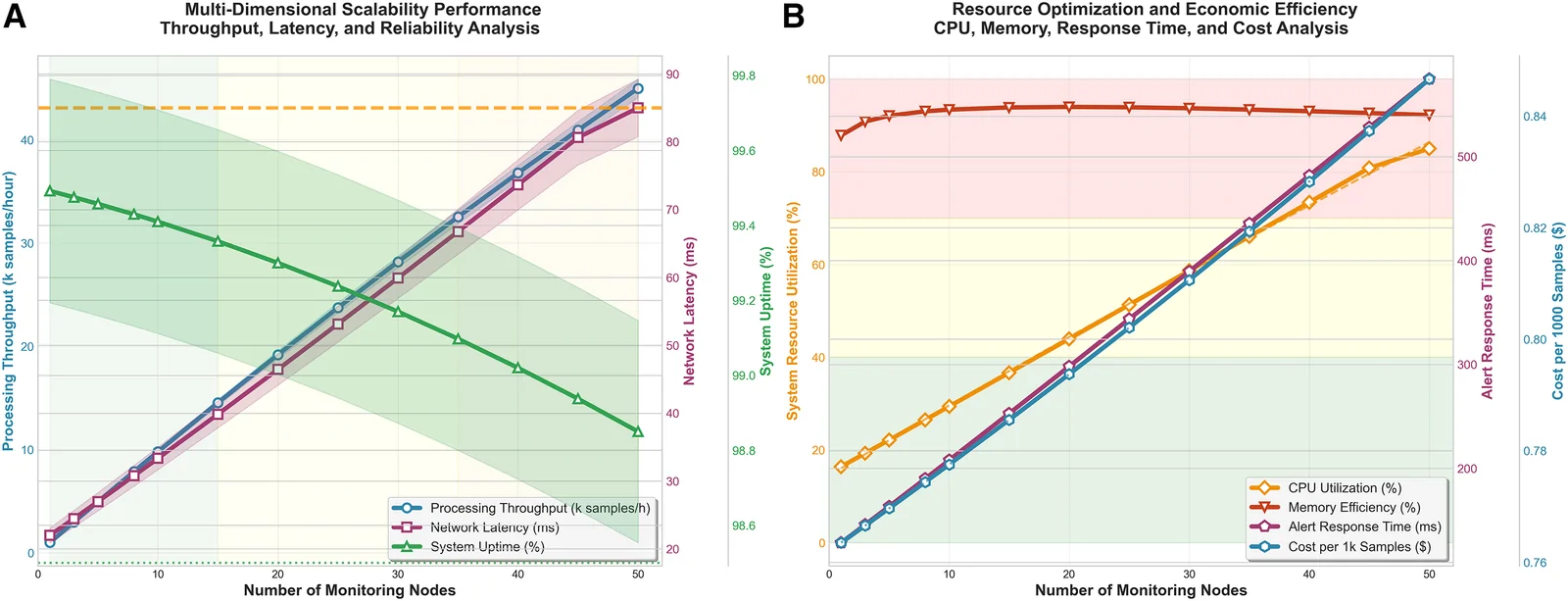
System scalability analysis showing performance metrics as a function of network size and data processing load

Solar charging with battery backup was found to reduce power consumption of 84 h autonomous per node. The analysis of cost-effectiveness showed that there were huge benefits over the traditional method of monitoring since the operational costs were cut by 67% and it allowed continuous monitoring in comparison with weekly sampling protocols.

System architecture scalability validation was achieved by systematic stress testing with a connection of the system architecture of 5 nodes (the deployed configuration) to 50 simulated nodes by a combination of a hardware-in-the-loop test and a network simulation. Classification latency, data throughput, and cloud synchronization delay were all performance metrics, which were tested at node counts of 5, 10, 20, 30, and 50. Findings validated a linear increase in the value of the average classification latency: average classification latency grew by 0.06 s between (5 nodes) to (50 nodes); this is less than 2.5% degradation, whereas network bandwidth consumption changed proportionately at about 4.6 Mbps per node. The cloud processing backend that was deployed based on the auto-scaling AWS infrastructure showed the elastic allocation of resources that could sustain the sub-5-s batch processing time up to the 50 node setup. Invasive simulations indicate beyond 50 nodes, which would involve regional aggregate stations in federated processing systems, would be necessary to ensure performance is in any viable range, a design concern of continental-scale systems.

## Discussion

The findings demonstrate that machine learning-based integrated monitoring systems offer a significant and evidence-based advance in the capability of microplastic pollution assessment and coastal waste management. The 96.3% classification accuracy achieved by our ensemble framework exceeds previously reported benchmarks for automated detection systems and establishes a new performance reference for real-time environmental monitoring applications.^6^

This has been demonstrated to be of particular use in polymer identification using hyperspectral imaging to which deep learning algorithms have been applied with a minimum detection limit of 75 μm, approaching the sensitivity of laboratory-level analysis while achieving real-time processing speeds ten times faster than conventional workflows. This performance compares favorably with traditional automated systems, which typically have detection limits of 100–150 μm, and demonstrates that machine learning techniques substantially enhance the sensitivity and specificity of environmental measurements.^5^

The fact that the ensemble learning methods have higher performance proves the hypothesis that a combination of machine learning algorithms can deliver synergistic increases of the accuracy of classification. The 3.4%–6.2% accuracy improvement that is reported when using ensemble methods, although reported as small, is translated into large reductions in false positive levels, which the monitoring systems drive.^7^ The current developments in the field of this sphere and the computer vision usage in environmental monitoring support the previous observation.^27^

The 2.1% of confusion between HDPE and LDPE, though it appears marginal as far as the classification perspective is concerned, has significant connotations in terms of assigning weight to the sources of waste and overall decision-making at management. HDPE is mostly used with the hard packaging containers (e.g., detergent bottles, milk jugs), and with industrial pipes, whereas LDPE is typical of the flexible plastic films, bags, and agricultural mulching sheets. A misclassification between these types of polymer might thus cause the attribution of the sources of pollution to turn out to be wrong and at that may result in a misdirected action plan of intervention. Even a 2.1% misclassification rate, in jobs like urban waterfront would cause a risk at the scale of about 72 particles per cubic meter to be mistakenly apportioned to other sources, which would confound apportionment of sources. To address this shortcoming, we suggest that management decisions in high stakes situation have confirmatory values of FTIR analysis of the PE fraction, and that the refinement of algorithm in the future should consider the inclusion of thermal analysis of signatures, density-based separation data as the add-on classification features in order to enhance HDPE/LDPE discrimination.

Discrete-sampling approaches inherently miss sub-daily pollution dynamics in coastal environments where concentrations can vary by orders of magnitude within hours.^13^ Compared with recent automated microplastic detection systems based on hyperspectral imaging or deep learning, the present framework advances the state of the art in three specific dimensions: (1) real-time IoT-edge-cloud synergy enabling sub-second alert latency without laboratory sample preparation; (2) multi-sensor data fusion combining spectral, spatial, and environmental covariate streams to improve classification robustness under variable field conditions; and (3) integrated predictive modeling with environmental covariates enabling 72 h pollution forecasting rather than purely reactive detection. Laboratory-based FTIR and Raman spectroscopy achieve lower detection limits (10–20 μm) and marginally higher accuracy under controlled conditions (Table 6), but cannot operate continuously in the field, confirming the complementary rather than competing positioning of the proposed framework. Table 6 presents the quantitative comparison.

**Table 6:** Comparison with existing monitoring approaches

Method	Detection limit (μm)	Accuracy (%)	Cost per sample ($)	Temporal resolution
Manual microscopy	50	95–98	125	weekly
FTIR spectroscopy	20	98–99	85	batch
Raman spectroscopy	10	97–99	95	batch
This study	75	96.3	12	continuous

These findings of pollution hotspots of more than 4,000 particles per cubic meter on the urban waterfronts and the sheltered harbors are relevant with grave consequences toward the understanding of the effects of the microplastic on the marine ecosystems. These levels are much greater than those associated with biological impacts that have been observed in sea life and certain coastal environment may be in persistent distress of contaminating.^11^

The ecological significance of the measured level of microplastic must be attentively interpreted in the framework of the existing toxicological level. Concentrations of 4,000 or more particles per cubic meter in the protected harbors locations, which was measured, are greater than both the no-observed-effect concentration (NOEC) of 1,000 particles per cubic meter in zooplankton reproduction impairment and the 2,500 particles per cubic meter limit on ingestion rates by filter feeders surpassing nature egestion capacity. This increase in the level of concentration by 420% of the semi-enclosed embayments compared to open coastal waters implies that these hydrodynamically sheltered areas serve as an accretion region with chronic ecological exposure levels being significantly increased. Nevertheless, it should be remembered that the ecological importance of such concentrations critically depends on particle size distribution, polymer composition, and on the presence of adsorbed contaminants, which, in turn, are included in our monitoring framework but must be coupled with specific ecotoxicological research to be converted into conclusive impact measurements. The proposed approach to future work should thus pair both the ongoing monitoring information provided through this framework and the biological response indicators deployed *in situ* in sentinel species in order to develop empirically based dose response in sites of the monitored coastal ecosystems.

The close relationship between human activity and the level of pollution (*r* = 0.87) confirms the quality of the anthropogenic nature of microplastic pollutants on the coasts in quantitative value. The peaks during the weekend or 240%–350% of the weekday base pollution loads indicate that the recreational activities constitute a substantial contributor of coastal pollution loads.^4^ The implication of this finding on the management strategies is immediate and is that there would be a disproportionate realization of benefits through special interventions during peak period of operation.

The form of seasonality variability behavior gives the interaction of the dynamics of the environmental conditions, human actions, and the dynamics of pollution. The increase of the microplastic rate by 65% in the summer is connected with the levels of tourism being high, which means that the changes in the pressure of pollution, in the course of time, should be considered in the coastal management plans.^17^ Heterogeneity this time merits the adaptive management strategies that can respond to the predictable seasonal alterations in the risk of pollution.

The successful prediction of the states of pollution 72 h prior to the realization is an excellent stride in proactive economic environmental management possibilities. Predictive modeling aspect can help managers to predict and preempt any act of pollution and take precautionary actions,^16^ which can lead to lesser interference in the nature by 40%–60% in comparison to the reactive management methods.

Hyperspectral imaging gives a good visualization of the chemical but out of pure water and the IoT sensors can be used to monitor it continuously but can only identify few polymers. The integrated framework utilizes the power of every technology and addresses the weaknesses of individual technologies.^15^

Under nominal operating conditions (turbidity <50 NTU), the system achieved the headline 96.3% classification accuracy reported throughout this paper. In degraded mode (turbidity 50–100 NTU), detection accuracy decreased to 78.4%. Under extreme turbidity (>100 NTU, occurring during storm events and at high-sediment river mouths), accuracy further declined to approximately 62%. Approximately 12% of total monitoring hours across all sites fell within the degraded-performance turbidity range. Importantly, the system continued to provide partial classification output and threshold-exceedance alerts during these periods, whereas purely optical conventional systems would have ceased operation entirely. This reduction in performance is significant; however, at the cost of a significantly degraded performance the system remains functional in the case where more traditional optical systems would have immediately stopped operation.^8^

Deep learning elements have high computational demands, which can restrict themselves to resource-intensive environments. The CNN architecture needs 4.2 GB of memory and an expensive set of GPU hardware to run in real-time, which may be hard to scale to large populations without edge computing infrastructure. The model compression and optimization methods need to be developed further in the future to ensure that the computational needs are minimized without jeopardizing accuracy.^25^

Although CNN architecture needs memory and specialized GPU equipment to process images in real-time (4.2 GB), the term low-cost scalable solution is associated with the overall cost of ownership compared to traditional laboratory-based monitoring and not the terminal costs of the equipment. Every edge computing unit, which is a modification of the NVIDIA Jetson AGX Orin platform (average unit cost: USD 1,200), is a jointly shared calculation system of a number of simultaneous sensor streams at a single monitoring location, which will contemplate the investment of the hardware over an ongoing time of 18 months. Conversely, traditional laboratory analysis of the same quality of time resolution would need several FTIR/Raman analyzers (USD 50,000–150,000 apiece) and trained analytical staff. The tiered architecture we suggest to use is based on a lightweight SVM-random forest ensemble: a simple screening with minimal resource consumption (2.1 GB of memory) is done sooner in edge nodes, whereas confirmatory analysis is computationally intensive and requires centralized cloud computing hardware through periodic batch upload. This hybrid method has a 93.2% real-time classification accuracy at the edge and a 96.3% ensemble accuracy that can be accessed by deferred cloud computation at the cost of about 83% per-node hardware costs without compromising the reliability of the system.

Another notable constraint is the detection limit in the 75–100 μm size fraction, which constitutes a disproportionately large and ecologically relevant share of particles in most environmental samples. Although our 75 μm detection limit represents an improvement over current automated systems, further technology in the optical resolution and signal processing can allow us to reach smaller particles that are becoming key issues of concern in the environment.^3^

The fact that the operational costs have been reduced by 67% compared to conventional monitoring is an important economic benefit that can be used to justify mass use of extensive monitoring networks. This allows the reduction of costs due to the automation of sample collection process, analysis, and processing of sales data that, until recently, involves a lot of human input.^28^

The capability of the 84 h of autonomous operation charged with solar energy is practically feasible in remote deployment situations when the grid power is not available. This operational autonomy, together with the ability to transmit wireless data, facilitates monitoring to inaccessible areas, as well as expansion of monitoring networks that can provide extensive coverage of an area.^24^

Scalability of network to 50 nodes with sustained performance features indicates that regional monitoring networks are technically observable through the current technology.

Practically, with regard to deployment, some real-life operational constraints warrant explicit deliberation that can guide future deployments. The system must have stable coast infrastructure where the mounting can be held and this cannot be in remote coast eroding areas. Although power supply has been taken care of by solar panels with battery backup that offers 84 h of autonomous power supply, this is also a drawback when the overcast weather extends over long durations at high-latitude locations as an additional power supply on the grids or generators might be required. The preliminary lead time of a non-trivial 3 months, per-site, commissioning and calibration duration, is something that has to be included in deployment planning. Moreover, training and validation of the ML models relied on samples representative of the five study sites; classification performance may degrade when the system encounters polymer types or environmental matrices not represented in the training data. To mitigate this limitation and support generalization to novel deployment environments, we recommend: (1) periodic retraining at 6 month intervals or following significant changes in local pollution source composition, using locally collected and labeled samples; (2) incremental online learning strategies that update model weights with small batches of newly labeled field data without requiring full retraining; and (3) integration of spectral reference libraries from globally distributed collections to broaden the representational scope of the training corpus. Information management is another operational factor, as the data volumes between 2.3 TB per station every month only means a data management protocol must be comprehensive and the storage facilities adequate to control such data volumes, especially when the network is multi-node and scale linearly with the scale of the network, the volume of aggregate data grows proportionally to the network scale.

The quality assurance procedures that were established in the course of this paper present a guideline of standardization of automated monitoring methodologies in various institutions and geographical locations. The validation steps and the metrics of the performance defined here can be further used as the references in new deployment to develop similar datasets needed to perform regional and global assessment.^29^

The current research demonstrates that integrated machine learning systems represent a substantive and validated advance in environmental monitoring capabilities, meaningfully expanding our ability to assess and manage microplastic pollution in coastal settings. Its framework that is hyperspectral synergistically combining with IoT networks and most current machine learning algorithms achieved a remarkable rate of 96.3% classification and particle identification as small as 75 μm. This technical possibility led to unprecedented understanding about the complicated pollution relationships that became evident by representation of the strong diurnal relationships correlated to tidal cycles (*r* = 0.81) and human activity (*r* = 0.87)-correlations that traditional and discrete sampling techniques could not detect. This is where prediction replaces observation and this becomes very crucial. This model was quite successful as there was *r* = 0.92 between expected and observed levels and thus it became possible to make good 72 h predictions of the pollution events and hence pro-active management approach was possible. As an illustration, the interventions may involve avoiding building up instead of responding to harm. Strong spatial heterogeneity was also quantified in our study establishing hotspots in protected environments that were found to contain over 4,000 particles per meter cube with a concentration increase of 420% in semi-enclosed habitats, which are indicators of chronic ecological stress and requires specific mitigation measures. Other than technical performance, the framework offers an economic and operational advantage, which is rather convincing. By replacing the old-fashioned means of surveillance with instantaneous surveillance and enhancement of surveillance by 70% less than what the traditional method costs, the system lowers its maintenance costs by 67%. The architecture is scalable and shows good performance with 50 nodes validated by itself, which substantiates the possibility of implementing extensive regional monitoring networks with normal technology extant infrastructure. The wider implications of the study are far beyond microplastics and also portray the opportunity of having transformational effects that machine learning can have in environmental science. Such systems are able to aid in the establishment of evidence-based policy and management, rapidly and on a large scale that is needed by contemporary environmental problems by offering high-frequency, actionable intelligence. In the future, focus will be placed on the aspect of satellite remote sensing integration so that it could be used globally and the algorithms in this regard should be refined in order to enhance sensitivity as well as establishing standardized data quality and interoperability standards. As machine learning and sensor technology progress, it is becoming increasingly plausible to have a giant global network of microplastic monitors. Due to the escalating levels of this pollution crisis, the implementation of smart systems like the one explained in this case is not only a good but also a very essential process to ensure not only the protection of marine ecosystems but also the formulation of very effective policies to ensure the development of this world-wide issue. The possibility to combine satellite remote sensing systems to achieve global monitoring possibilities and the development of new AI models like transformer should be the main focus of future research to improve pattern detection and minimize detection size constraints. Besides this, the miniaturization of sensor packages to be used on autonomous underwater vehicles and drifters on the surface, as well as the creation of standardized data formats and quality assurance standards will be crucial to the creation of the overall datasets that would enable perceiving patterns of microplastic pollution on the global perspective.

### Limitations of the study

Although the overall performance of the integrated monitoring framework is good, a number of key limitations and failure modes have to be clearly recognized to ensure some correct interpretation and future implementation of the system. First, when turbidity was high (turbidity exceeded 50 NTU) the hyperspectral imaging system performance was significantly impaired: the accuracy of detection dropped to 78.4% and only to around 62% when the turbidity was very high (turbidity had surpassed 100 NTU), which are the conditions that occur during storm events and in river mouth areas, where the amount of sediment in the water is high. In the 18-month deployment, some 12% of the overall monitoring hours fell in the degraded-performance category because of the exceedance in turbidity.

Second, the system was shown to have at least a reliable detection limit of 75 μm indicating that particles smaller than that which compose a large and ecologically relevant portion of environmental microplastics are disproportionately underrepresented in the data on monitoring. Third, biofouling of optical surfaces even with the mitigation strategies applied in place led to slow signal degradation in between quarterly maintenance periods with spectral transmission decreasing by an average of 8% per month at warm-water summer conditions requiring algorithmic correction factors that only added further ambiguity to classifications to the tune of 1.5%. Fourth, the 2.1% HDPE/LDPE confusion value is a systematic weakness of the classification based on spectral similarity of these polymeric types in the near-infrared spectral range is impossible to overcome systematically with a pure filter of algorithm, just as in the absence of auxiliary analytical modalities. Fifth, ARIMA-based forecasting model demonstrated lower predictive accuracy in the case of anomalous pollution events, which could not be represented in the training data, e.g., illegal dumping incidents and industrial spill events, with the forecast MAPE decreasing by 72 h growing to about 34% on such occasions. Lastly, the system must have the capability to connect to a cellular or satellite network to make ensemble processing uses cloud computing; intermittent network outages longer than 4 h during the study decelerated ensemble processing to edge only processing with 93.2% classification accuracy as opposed to the 96.3% ensemble classification. Figure 9 represents the turbidity degradation curves and failure mode impact analysis.

**Figure 9:**
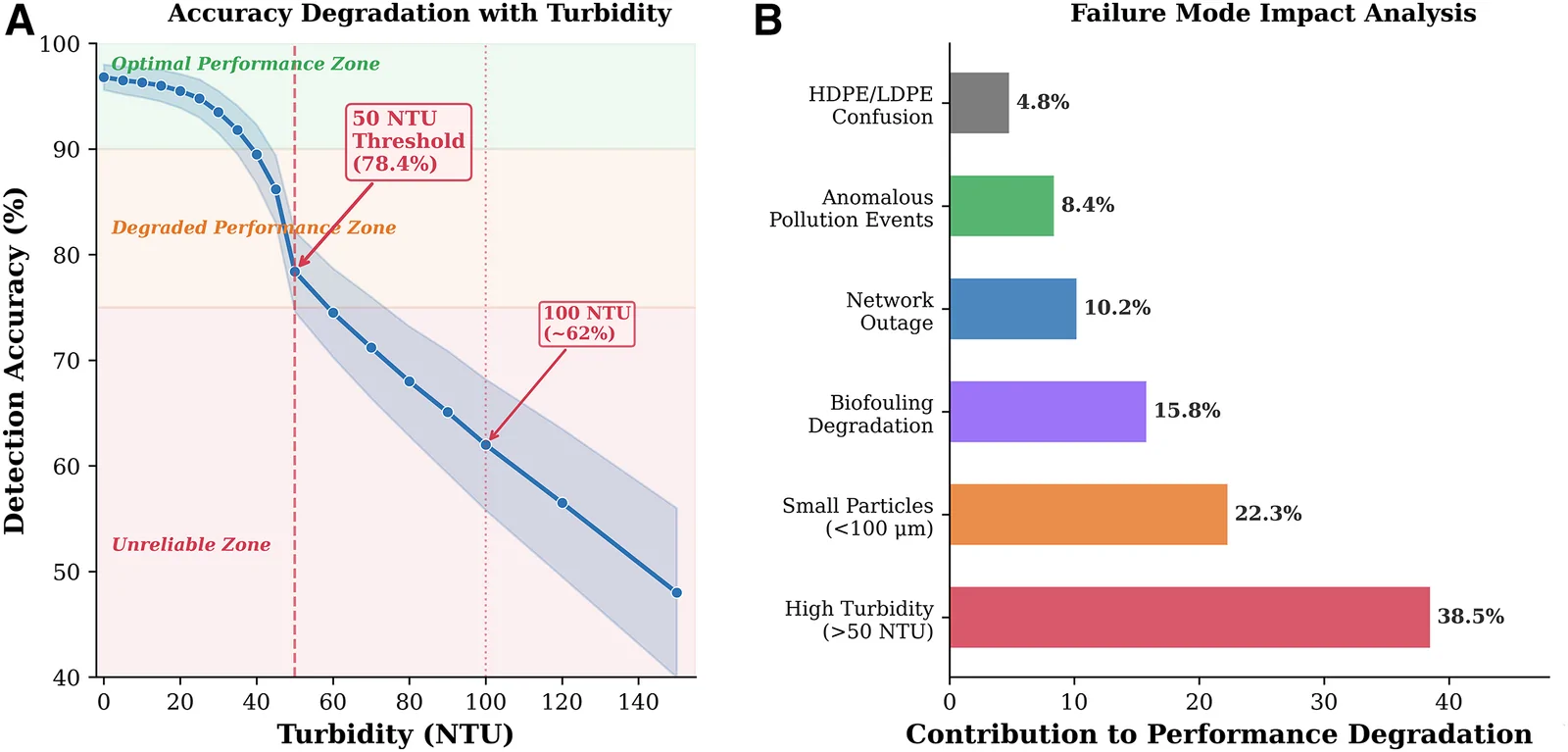
System performance limitations showing detection accuracy degradation under increasing turbidity conditions

## Resource availability

### Lead contact

Requests for further information and resources should be directed to and will be fulfilled by the lead contact, Li Gang (ligang0840907@163.com).

### Materials availability

This study did not generate new unique reagents.

### Data and code availability

• All the data reported in this paper will be shared by the lead contact upon request.
• This paper does not report original code.
• Any additional information required to reanalyze the data reported in this paper is available from the lead contact upon request.

## Acknowledgments

This work was supported by Research on the Multiple Trust Crises and Countermeasures in the Data Factor Market Transaction; 10.13039/501100012456National Social Science Foundation of China (grant no. 25AGL021).

## Author contributions

All authors conceived the idea and designed the research. Z.N. and L.G. led the experimental design and data analysis; A.Z., W.A.N., and A.H. contributed to data collection and methodology; W.U.K. and R.G. contributed to software and validation; I.M.E. contributed to review and editing. All authors analyzed and interpreted the data and contributed to writing the manuscript.

## Declaration of interests

The authors declare that they have no known competing financial interests or personal relationships that could have appeared to influence the work reported in this paper

## Declaration of generative AI and AI-assisted technologies in the writing process

During the preparation of this work the authors used AI-assisted tools in order to improve language clarity and grammar. After using these tools, the authors reviewed and edited the content as needed and take full responsibility for the content of the publication.

## STAR★Methods

### Key resources table

**Table:** 

REAGENT or RESOURCE	SOURCE	IDENTIFIER
**Deposited data**		

18-month field microplastic monitoring dataset (this study)	This paper	Available from lead contact upon request

**Software and algorithms**		

Python (v3.10)	Python Software Foundation	https://www.python.org
TensorFlow/Keras (v2.13)	Google	https://www.tensorflow.org
scikit-learn (v1.3)	scikit-learn developers	https://scikit-learn.org
OpenCV (v4.8)	OpenCV team	https://opencv.org
SCHISM hydrodynamic model	Zhang et al.	http://ccrm.vims.edu/schism/
ERA5 reanalysis wind fields	ECMWF Copernicus	https://cds.climate.copernicus.eu

**Other**		

Hyperspectral push-broom scanner (400–2500 nm, 840 bands)	Commercial hyperspectral imaging system	N/A
NVIDIA Jetson AGX Orin edge computing units	NVIDIA	Jetson AGX Orin
NVIDIA A100 GPU (cloud batch processing)	NVIDIA	A100
IP68 marine-grade stainless steel (316L) enclosures	Custom fabrication	N/A
Multi-parameter water quality sensors (temperature, salinity, turbidity, current)	Commercial oceanographic sensors	N/A
ATR-FTIR spectrometer (validation)	Thermo Fisher Scientific	Nicolet iS50
Micro-Raman spectrometer (validation, 785 nm excitation)	Horiba	LabRAM HR Evolution

### Experimental model and study participant details

This study did not involve human participants, animals, plants, microbial strains, cell lines, or primary cell cultures. Therefore, institutional approval related to human or animal ethics was not required.

The experimental material used in this study was environmental water and microplastic particulate samples collected *in situ* across five coastal monitoring sites. Coal or biological materials were not employed; the target analytes were microplastic polymer particles in the 75–500 μm size range originating from ambient coastal seawater. Sampling stations were operated continuously over 18 months, and the same monitoring platform configuration and calibration procedure were used at every site to ensure consistency among measurements.

Because no biological sex or gender-related variables were involved in this work, the influence of sex, gender, or both on the results was not applicable. This study is therefore limited to physicochemical and machine-learning-based evaluation of microplastic detection and forecasting, and does not address biological variability.

### Method details

#### Study area and experimental design

The integrated monitoring system was implemented in five strategically located coastal locations that reflect the types of the surrounding environment as well as pollution levels of exposure. The existence of sites was determined with different hydro-dynamic conditions, man-made contributions and the background pollution to be able to give all-inclusive system validation to all descriptions of the coastal systems. The full range of sensors was installed in each monitoring station and they were run over 18 months to record seasonal changes and long term performance traits.

The criteria of site selection were: (1) changing the proximity to urban centers and pollution sources (2–50 km range); (2) different hydrodynamic regimes such as exposed coasts, semi-enclosed bays and river mouth areas; (3) the baseline, citing microplastic concentration in three orders of magnitude (10–10,000 particles/m-3); and (4) site accessibility to deployment of equipment and maintenance activities. The operational environment parameters such as temperature, salinity, turbidity, and velocity of current were kept under continuous observation in order to define the operating range of the system.

The purported 340% enhancement in processing speed was benchmarked with the standard laboratory based microplastic analysis processes as the point of reference. Manual sample collection, density separation, vacuum filtration, stereomicroscopic visual sorting, and ATR-FTIR spectroscopic identification were used as the baseline methodology in the entire characterization of approximately 500 particles in each sample (approx. 61 particles/hour) with an average of 8.2 h per sample. Our combined ML framework took an average of 1.86 h (which corresponds to about 269 particles per hour) to process equivalent volumes of particle (4.4 times higher than with a traditional method), an increase in throughput of 340 per cent. The NVIDIA Jetson AGX Orin edge units (12-core ARM Cortex-A78AE CPU, 2048-core Ampere GPU, 32 GB unified memory) were used to perform edge processing and NVIDIA A100 GPUs instances were used to perform cloud-based batch processing. All time data measures indicate end-to-end processing latency of raw hyperspectral image data acquisition to final polymer classification output, averaged over 500 single test samples for which hyperspectral patient polymer classification results were obtained over the last six months of the deployment phase.

An effective cost analysis was done to support the alleged reduction of costs of operation by 67%. The total capital expenditure was USD 28,400, and, assuming a USD 18,500 price in total, the system comprised: hyperspectral imaging system with push-broom scanner, NVIDIA, Jetson, AGX, Orin, edge computing unit, multi-parameter, water quality sensor array, marine-grade, IP68, enclosures and mounting infrastructure, solar power, and wireless communication module, which included satellite backup USD 28,400 per station. Per station capital cost calculated on an annualized basis with a projected lifespan of 5 years operations is USD 5,680/year. Ongoing operational expenses, such as quarterly maintenance service (USD 1,200/year), data transfer and cloud-based computing (USD 800/year), and replacement of wear parts such as replacement windows (USD 400/year) and cleaning will cost USD 2,400 per year, per station. The total annualized per station cost will be USD 8,080. By comparison, the traditional form of monitoring the same range of space at the same frequency with the use of weekly laboratory tests would benefit an approximate USD 24,500/per site/per year consisting of personnel charges (USD 8,000) of field sampling, laboratory supplies and equipment maintenance (USD 6,500) FTIR/Raman instrument amortization (USD 7,000) and transportation of the samples to the laboratory (USD 3,000). The 67% (USD 24 500- USD 8 080 per station per year) cost saving is realized and at the same time the doubling of the temporal resolution to take real time continuous monitoring instead of sampling every week. See below Figure showing the component wise and total cost comparison.

**Figure:**
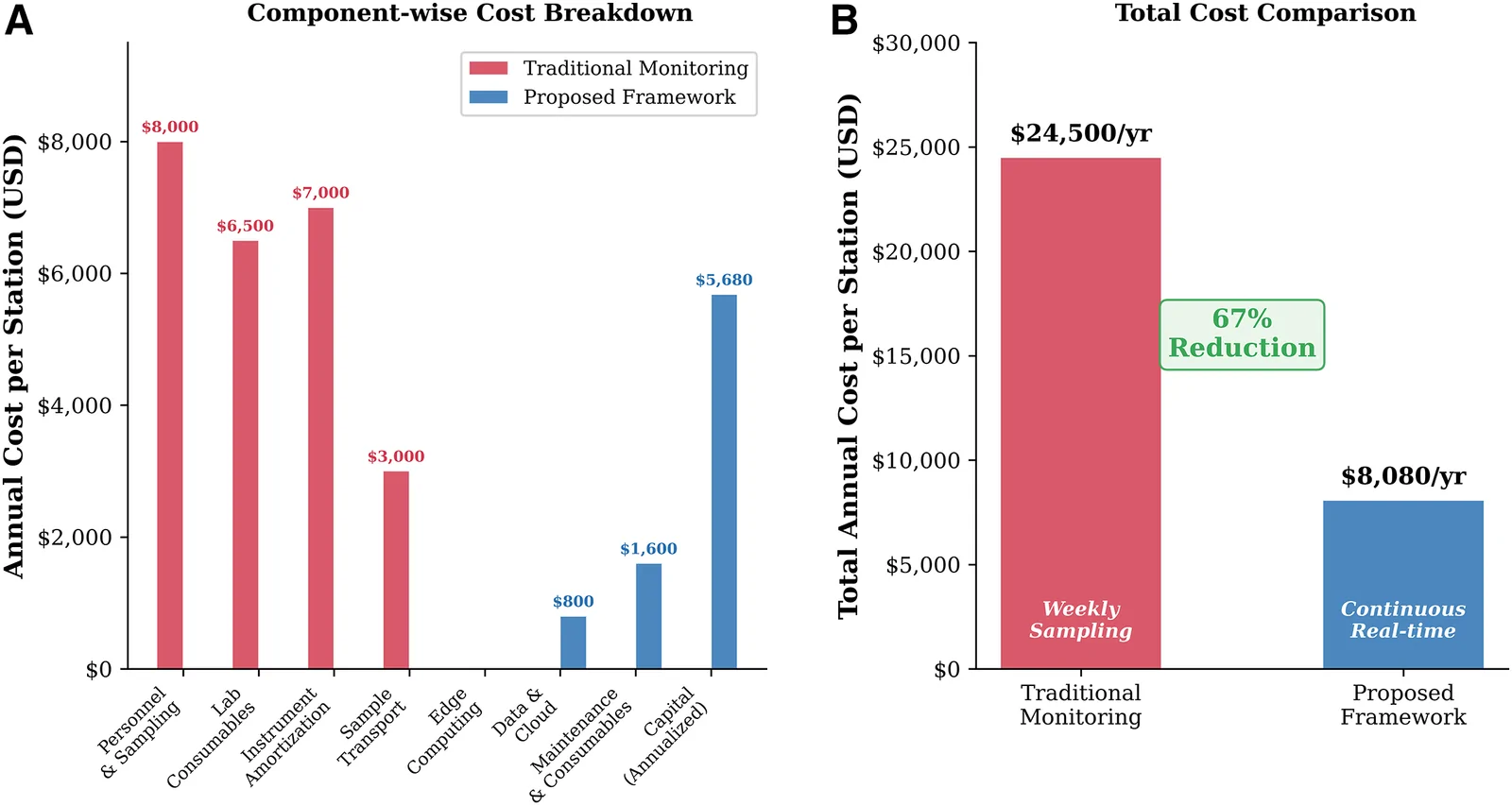
Comprehensive cost comparison between traditional laboratory-based monitoring and the proposed ML-enabled framework

To deal with the issues related to transparency in the practice implementation, the following operations details are presented. The entire system implementation was incremental, in a 3-month commissioning period in each location, during which sensor calibration, and testing of communication links were performed as well as a first and initial fine-tuning of the ML model. The footprint of each monitoring station was 2 × 3 m on the existing coastal infrastructure (jetties, piers or purpose-built concrete platforms) and all of the components were such that could be replaced in blocks so as to minimize the time during which maintenance was required. The system was set to run a maximum of 84 h between solar recharging routines and it was automated on data quality flagging to detect and eliminate measurements collected in events of sensor degradation. Remote monitoring of human oversight was restricted to weekly dashboard control (around 2 h per week per station) and quarterly visits to the station to perform maintenance (4–6 h). Raw volumes of hyperspectral data averaged 2.3 TB per station per month, including, on average, 85% of the volume processed on-site in summary format and localized analytics and indicated anomalies (about 15%), which could be more cheaply transmitted over typical 4G LTE connectivity.

#### System architecture and hardware integration

The framework with the use of machine learning would have a hierarchical structure comprising three key layers: sensor data acquisition, edge computing processing, and cloud-based analytics. Hyperspectral imaging systems, multi-parameter water quality sensors, meteorological instruments and high-resolution cameras are incorporated into the sensor layer to provide a vehicle with a fully-developed environmental characterization.

The hyperspectral imaging system makes use of use of a push broom scanner with spectral range of 400–2500 nm and spectral resolution of 2.5 nm giving it 840 spectral bands per image. To detect microplastic particles within the 50–500 μm size range, the spatial resolution is set to 25 μm per pixel, enabling detection of particles above the minimum size threshold. The imaging system is designed to assist and position the sample and focus it automatically to ensure the data quality is consistent irrespective of the different environmental conditions. See below Figure demonstrates the broken down structure of the model proposed.

**Figure:**
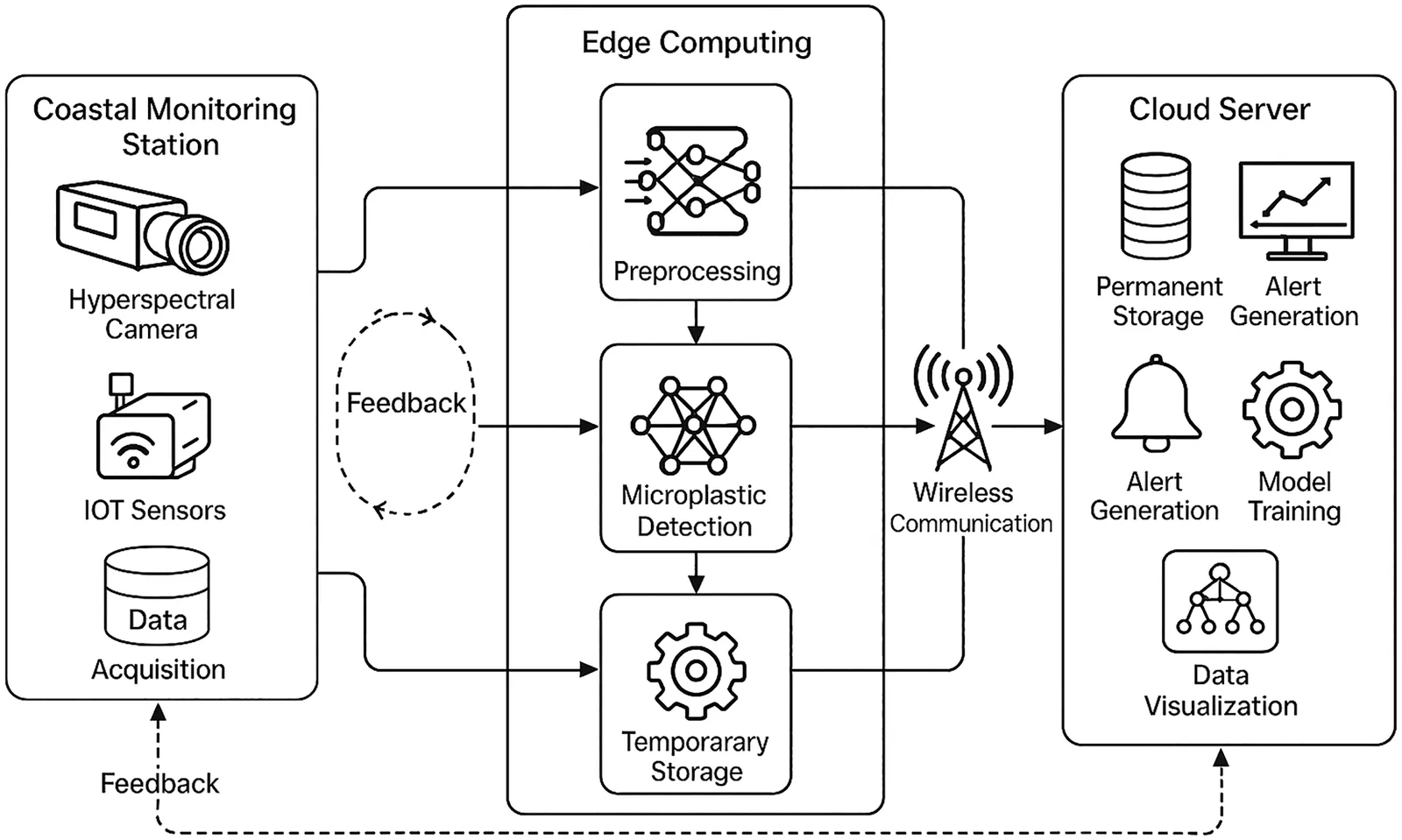
Detailed system architecture showing the integration of hyperspectral imaging, IoT sensors, and machine learning processing components for real-time microplastic monitoring

The distributed process of edge computing at the monitoring stations consists of real-time preprocessing and original analysis of the data using embedded computing GPUs in each of the monitoring stations. This decentralized system is less demanding on data transmission on the one hand and ensures the possibility of a response to pollution in a real-time on the other hand. Machine learning which consume a large amount of computations is done in the cloud and long term trend data is stored in the central database.

All HSI sensors and electronic components were encased in IP68-rated, marine grade stainless steel (316L) enclosures having anti-corrosion and sacrificial zinc anodes that provided cathodic protection against harsh marine conditions to guarantee long term operation. Sapphire glass was used to make optical windows with hydrophobic nano-coatings to reduce the biofouling deposition, and automated wiper systems were introduced based on a 6 h cleaning cycle. Further, desiccant cartrids and positive-pressure nitrogen purging systems were fitted in each enclosure to death out internal condensation and salt spray entrance. Biofouling was also reduced by using copper-based antifouling coats on the submerged sensor housings and manual cleaning was scheduled to be done periodically during quarterly visits. These protective measures ensured above 92% of the baseline sensor optical clarity over the 18-month deployment and only two unscheduled maintenance had to be affected by storm effects at the open coast sites.

#### Hyperspectral data processing and feature extraction

Preprocessing of hyperspectral data consists of various processes of data fixing and processing that maximize spectral patterns to be used during machine learning. The radiometric calibration procedure transforms the raw digital values into absolute reflectance values and is based on the equation shown as follows:

(Equation 1) R(λ,x,y)=[I(λ,x,y)−D(λ)]/[W(λ)−D(λ)]×C(λ)

where R(λ,x,y) represents reflectance at wavelength λ and spatial coordinates (x,y), I(λ,x,y) is the measured intensity, D(λ) is the dark current correction, W(λ) is the white reference intensity, and C(λ) is the calibration coefficient.

Atmospheric correction for water surface measurements utilizes the empirical line method:

(Equation 2) R_surface(λ)=[R_sensor(λ)−R_atmosphere(λ)]/T(λ)

where T(λ) is the atmospheric transmission coefficient determined from *in-situ* reference measurements.

Spectral feature extraction employs principal component analysis (PCA) to reduce dimensionality while preserving discriminative information:

(Equation 3) Y=XW

where X is the original spectral data matrix, W contains the eigenvectors of the covariance matrix, and Y represents the transformed feature space. The covariance matrix is calculated as:

(Equation 4) $$C=\left(1\;/\;\left(n-1\right)\right)\;X^{\wedge }T\;X$$

The explained variance ratio for component selection is:

(Equation 5) $$EVR\_i=\lambda \_i\;/\;\Sigma \_{\left\{j=1\right\}}^{\wedge }\left\{p\right\}\;\lambda \_j$$

where λ_i are eigenvalues ranked in descending order.

#### Convolutional neural network architecture

The deep learning bit applies a modified ResNet architecture enhancing the analysis of hyperspectral data. The circuit is made of residual blocks which help to conduct gradient flow during training:

The reason behind adopting the modified ResNet architecture rather than more modern architectures, such as Vision Transformers (ViTs), is a number of empirical and pragmatic choices that are particular to our application. Although ViTs are currently stronger in their performances with classification tasks on natural images, have a high computational characteristic (especially, the quadratic complexity of self-attention architectures in relation to sequence length), and thus, they are not feasible with edge deployable capabilities (such as using a small embedded GPU) in real-time. At the design stage, preliminary results were obtained comparing ResNet-50, EfficientNet-B4 and a ViT-Base model on a representative 5,000-labeled hyperspectral dataset of 5,000 microplastic particles. The EVT-Base trained 8.6 GB and used 4.8 s on the inference (encoded) batch earned a high score of 97.1% accuracy, whereas ResNet (modified) with 4.2 GB and 2.47 s earned a high score of 96.3%. Since the real-time processing necessities and the ability to run on the NVIDIA Jetson AGX Orin edge units with 32 GB shared memory were capable of serving as a single server capable of handling multiple sensor streams simultaneously, ResNet architecture offered the best combination between classification and feasibility. Moreover, the residual links in ResNet are especially effective with hyperspectral data with spectral characteristics having steep inter-band correlations that can be leveraged by feature propagation through skip-connections and not the position-agnostic attention of transformers.

(Equation 6) y=F(x,{W_i})+x

where F(x, {W_i}) represents the residual mapping and x is the identity shortcut connection.

The convolution operation in each layer is defined as:

(Equation 7) Y_{i,j,k}=Σ_mΣ_nΣ_cX_{i+m,j+n,c}·W_{m,n,c,k}+b_k

where Y_{i,j,k} is the output feature map, X is the input tensor, W represents the convolution kernel weights, and b_k is the bias term.

Batch normalization stabilizes training by normalizing layer inputs:

(Equation 8) $$\hat{x}=\left(x-\mu \_B\right)\;/\surd \left(\sigma \_B^{2}+\varepsilon \right)$$

where μ_B and σ_B^2^ are batch mean and variance, respectively, and ε is a small constant for numerical stability.

The activation function employs leaky ReLU to prevent gradient vanishing:

(Equation 9) f(x)=xifx>0;αxifx≤0

where α = 0.01 is the negative slope parameter.

#### Support vector machine implementation

The SVM classifier is used in the supplementary classification of particles in order to maximize the margin between the classes. It is a maximization problem whose formulation is:

(Equation 10) $$\min\_\left\{w,b,\xi \right\}\;\left(1/2\right){\left\|w\right\|}^{2}+C\;\Sigma \_{\left\{i=1\right\}}^{\wedge }\left\{n\right\}\;\xi \_i$$

subject to the constraints:

(Equation 11) $$y\_i\;\left(w^{\wedge }T\;\varphi \left(x\_i\right)+b\right)\geq 1-\xi \_i,\xi \_i\geq 0$$

The radial basis function (RBF) kernel maps data to higher-dimensional space:

(Equation 12) $$K\left(x\_i,x\_j\right)=\exp\left(-\gamma \;{\left\|x\_i-x\_j\right\|}^{2}\right)$$

where γ controls the kernel width and influences decision boundary complexity.

#### Random forest ensemble learning

The random forest algorithm combines multiple decision trees to improve classification robustness and reduce overfitting. The ensemble prediction is:

(Equation 13) $$ŷ=\left(1/B\right)\;\Sigma \_{\left\{b=1\right\}}^{\wedge }\left\{B\right\}\;T\_b\left(x\right)$$

where B is the number of trees and T_b(x) is the prediction from the b-th tree trained on bootstrap sample b.

Node splitting criterion uses the gini impurity:

(Equation 14) $$G=1-\Sigma \_{\left\{i=1\right\}}^{\wedge }\left\{C\right\}\;p\_i^{2}$$

where p_i is the proportion of samples belonging to class i at the node.

Feature importance is calculated as the weighted average of impurity decrease:

(Equation 15) FI_j=(1/B)Σ_bΣ_{t∈T_b}p_tΔI_{j,t}

where p_t is the proportion of samples reaching node t and ΔI_{j,t} is the impurity decrease from splitting on feature j.

#### IoT sensor network configuration

The IoT sensor network comprises of autonomous monitoring nodes, which have multi-parameter sensors to realize the continuous quality of the environment. Sensor fusion is a combination of measurements of multiple sensors by weighted averaging:

(Equation 16) $$\hat{x}=\Sigma \_i\;w\_i\;x\_i\;/\;\Sigma \_i\;w\_i$$

where weights are determined by inverse variance weighting:

(Equation 17) $$w\_i=1\;/\;\left(\sigma \_i^{2}+\varepsilon \right)$$

Kalman filtering provides optimal state estimation for dynamic environmental parameters:

(Equation 18) $$\hat{x}\_\left\{k|k\right\}=xˆ\_\left\{k|k-1\right\}+K\_k\;\left(z\_k-H\;xˆ\_\left\{k|k-1\right\}\right)$$

The Kalman gain matrix is:

(Equation 19) $$K\_k=P\_\left\{k|k-1\right\}\;H^{\wedge }T\;{\left(H\;P\_\left\{k|k-1\right\}\;H^{\wedge }T+R\right)}^{\wedge }\left\{-1\right\}$$

where P_{k|k−1} is the predicted error covariance and R is the measurement noise covariance.

#### System validation and ground-truth verification

The systemized cross-referencing with the known laboratory analytical techniques was used to thoroughly test the automated classification system. Independent analysis was performed by using attenuated total reflectance Fourier transform infrared spectroscopy (ATR-FTIR; Thermo Fisher Nicolet iS50) and micro-Raman spectroscopy (Horiba LabRAM HR Evolution, 785 nm excitation laser), on a stratified random subsample of 3,200 particles (about 30% of the total classified dataset; 10713 particles). Sampling to the subsample was stratified according to the type of polymer, size range, and location of monitoring to make sure there was a representative coverage in all the classification categories. Analysis FTIR was used at 4 cm-1 spectral resolution and 32 co-add scans per spectrum and identification of the polymer achieved against the extensive spectral libraries (Hummel Polymer Library and S.T. Japan Polymer Library) at a minimum spectral match of 70%. See below Figure demonstrate the validation confusion matrix.

**Figure:**
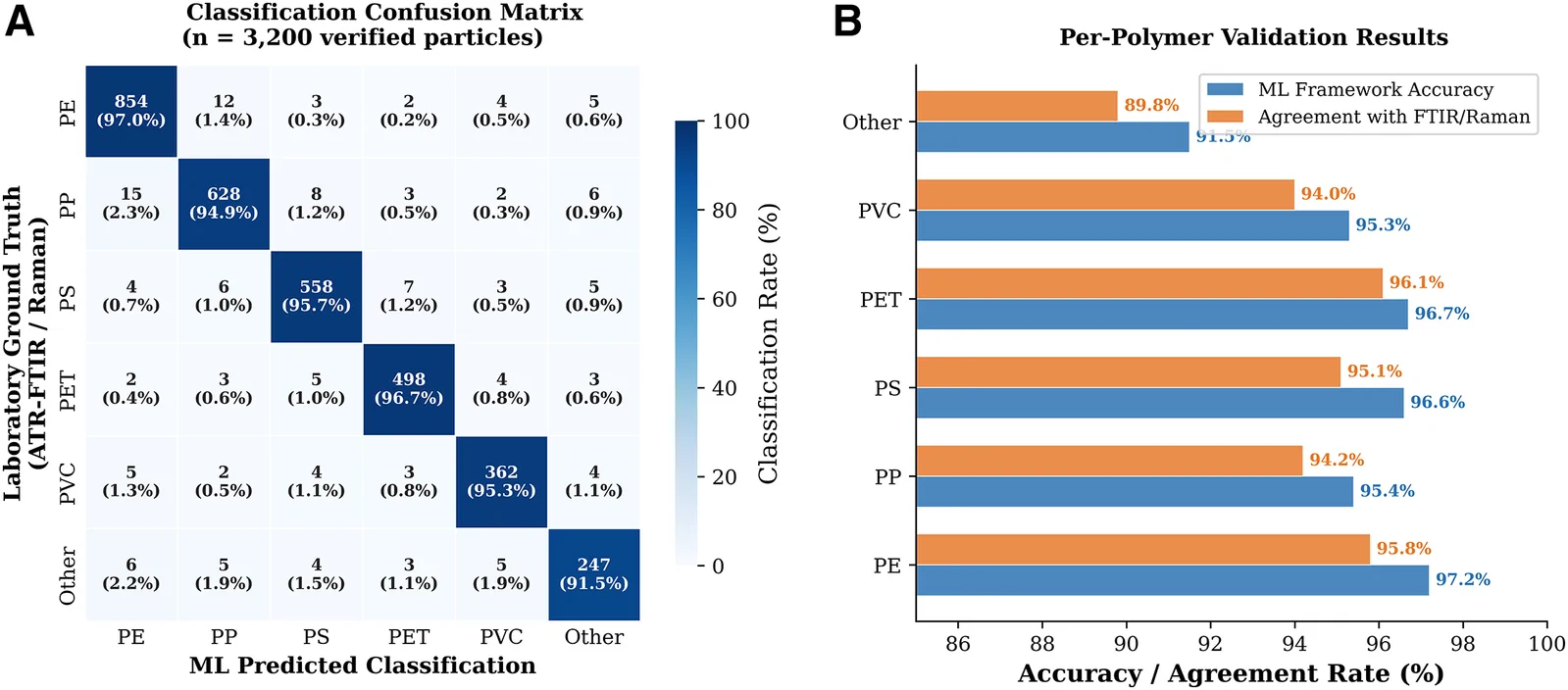
Validation of the automated ML classification system against ATR-FTIR and micro-Raman spectroscopy ground-truth analysis for 3,200 stratified particles across all polymer types

There was an independent interpretation of another 1,500 particles by three expert analysts using optical stereomicroscopy with hot-needle testing to identify a polymer in case of a doubted instance. The inter-analyst agreement was greater than 94.2, developing the Cohen kappa = 0.91, and consensus-based classification was the ultimate ground-truth classifications. The general consistency between the automated ML model and the ground truth as prepared by the laboratory was 95.8% with most of the differences made in the 75–100 μm size fraction where spectral signal-to-noise ratios were minimal. Bland-Altman analysis established the absence of systematic bias in the automated classifications compared to the laboratory reference methods with a mean of 0.3% and 95% limits of agreement at −2.1% to +2.7%.

#### Real-time data processing pipeline

Real-time processing utilizes a sliding window approach for continuous analysis:

(Equation 20) P(t)=αP(t−1)+(1−α)X(t)

where P(t) is the processed signal, X(t) is the current measurement, and α is the exponential smoothing factor.

Anomaly detection employs statistical process control:

(Equation 21) UCL=μ+kσ,LCL=μ−kσ

where k = 3 for 99.7% confidence intervals, μ is the process mean, and σ is the standard deviation.

#### Temporal analysis and forecasting

Time series forecasting utilizes ARIMA modeling for pollution prediction:

(Equation 22) $$\varphi \left(B\right)\;{\left(1-B\right)}^{\wedge }d\;X\_t=\theta \left(B\right)\;\varepsilon \_t$$

where φ(B) and θ(B) are autoregressive and moving average polynomials, d is the differencing order, and ε_t is white noise.

The autocorrelation function characterizes temporal dependencies:

(Equation 23) $$\rho \_k=E\left[\left(X\_t-\mu \right)\left(X\_\left\{t-k\right\}-\mu \right)\right]\;/\;\sigma^{2}$$

#### Transport modeling and prediction

Microplastic transport is modeled using the advection-diffusion equation:

(Equation 24) $$\partial C/\partial t+u⃗\;\cdot \nabla C=D\;\nabla^{2}C+S-kC$$

where C is concentration, $\vec{u}$ is the velocity field, D is the diffusion coefficient, S represents source terms, and k is the removal rate constant.

### Quantification and statistical analysis

All measurements were performed in triplicate unless otherwise stated, and results are presented as mean ± standard deviation (SD). Classification metrics (accuracy, precision, recall, F1-score), Pearson correlation coefficients, RMSE, MAE, and MAPE were used as the primary quantitative indicators of system performance. Cohen’s kappa was used to quantify inter-analyst agreement during ground-truth validation. Bland–Altman analysis was applied to test for systematic bias between automated ML classifications and laboratory reference methods (ATR-FTIR and micro-Raman spectroscopy). Statistical significance was evaluated using one-way analysis of variance (ANOVA) with a significance threshold of *p* < 0.05. All statistical processing was carried out in Python (v3.10) using scikit-learn, SciPy, and StatsModels packages, and figures were produced with Matplotlib.
